# How can general dental practitioners help in the management of sleep apnoea?

**DOI:** 10.1038/s41415-023-5684-1

**Published:** 2023-04-14

**Authors:** David Parmenter, Brian J. Millar

**Affiliations:** 41415171444001 Dental Core Trainee, Restorative Dentistry, King´s College Hospital, UK; 41415171444002 Clinical Professor of Dental Education and Consultant in Restorative Dentistry, Faculty of Dentistry, Oral and Craniofacial Sciences, King´s College London, UK

## Abstract

This article discusses the aetiology, prevalence and treatment of obstructive sleep apnoea (OSA) and highlights the important role general dental practitioners can perform in improving the quality of life of patients suffering from OSA. Clinical and laboratory stages of making a mandibular advancement appliance are also highlighted.

Members of the dental team have a duty of care to our patients. The earlier undiagnosed cases of OSA are referred for treatment, the less morbidity and potential mortality endured by patients.

After reading this article, the reader should have a greater understanding of OSA, how to identify symptoms of the condition in patients and be confident in referring patients to appropriate healthcare professionals.

## Key points

- Provides an overview of the aetiology, symptoms, risk factors and treatment of sleep apnoea.
- Documents the clinical and laboratory stages for creating a mandibular advancement appliance.
- Introduces the importance of a multidisciplinary approach in treating obstructive sleep apnoea.

## Introduction to sleep apnoea and its prevalence

Obstructive sleep apnoea (OSA) is the most common form of sleep-related breathing disorder.^1^ OSA is characterised by obstruction or narrowing of the upper airway during sleep when the throat muscles relax. Airway obstruction causes repeated absent or shallow breaths lasting for ten seconds or more.^2^ Sleep-related hypoventilation leads to hypoxaemia, eventually resulting in arousal from sleep.^3^This cycle is repeated multiple times throughout sleep.

OSA can affect people of all ages, but is more common in men than women and in overweight people of both sexes.^4^ Approximately 1.5 million adults in the UK suffer from OSA; however, only 15% of cases have been officially diagnosed. Untreated OSA can shorten life expectancy and result in serious comorbidities, including hypertension, diabetes, stroke and heart disease. Despite a variety of simple and effective treatments being available for OSA, only an estimated 330,000 adults currently receive treatment.^5^

A survey carried out by the British Lung Society found that nearly half of people who were aware they or their partner snored knew what OSA was. Furthermore, they estimate that the NHS could save £55 million a year if all moderate to severe cases of OSA were diagnosed and treated.^6^

### Symptoms

The core symptoms of OSA are excessive daytime sleepiness and dysfunction as a result of non-refreshing fragmented sleep, which has an overall reduction in quality of life.^7^ OSA patients often report witnessed episodes of absent breathing during sleep and have difficulty sleeping with their partner due to the volume of their snoring.^8^

### Risk factors

Age and obesity are the most significant risk factors for OSA. A body mass index of over 25 kg/m² has been found to have a 93% sensitivity for OSA and being over 65 increases the risk of developing the condition.^9^ Sleeping in a supine position encourages the tongue and soft palate to fall backwards onto the back of the throat, causing obstruction of the upper airway.^10^ Any anatomy that reduces functional space in the upper airway, such as a macroglossia, excess fat in the palate, or adenoidal tonsillar hypertrophy, predisposes young, healthy individuals to OSA.^2^

### Aetiology

During sleep, the upper airway relaxes. Pharyngeal dilator tone is lost and the base of the tongue and soft palate relax and rest onto the pharyngeal wall, resulting in a partial or complete airway obstruction, causing breathing to stop (apnoea). Hypoventilation causes a drop in blood oxygen levels, stimulating arousal in the central nervous system and an enhanced respiratory effort, which is observed as a gasp for air and restlessness. Depending on the severity of the individual's OSA, this cycle is then repeated multiple times throughout the night.^11^^,^^12^

### Diagnosis

The Epworth Sleepiness Scale (ESS) is a subjective questionnaire which measures daytime sleepiness by asking patients how likely they are to fall asleep during certain situations. The higher the score, the more severe the symptoms of daytime sleepiness.^13^The ESS helps differentiate between simple snorers and OSA; however, it is not diagnostic when used alone. The ESS is a subjective questionnaire at risk of bias or falsification of symptoms.^6^

Pulse oximetry and polysmography are objective, overnight sleep studies, which analyse breathing patterns and blood oxygenation and can be used to diagnose OSA.^5^ Pulse oximetry is the simplest way to confirm OSA, while polysomnography is reserved for more complex cases, or where simple tests prove inconclusive.

The Apnoea Hypopnea Index (AHI) score is calculated from data from an overnight sleep study and determines the severity of OSA. The AHI score measures the number of apnoeas and hypopneas per hour of sleep and is completed by the respiratory physician before referral. Five episodes of apnoea or hypopnea an hour is diagnostic for OSA, while a score of greater than 30 is diagnostic of severe OSA.^10^

## Management options

OSA treatment aims to improve an individual's quality of life by reducing daytime sleepiness and systemic health complications by reducing AHI scores. Behavioural changes such as; weight loss, smoking and alcohol cessation, modification of sleeping position from supine to side sleeping, are first line treatments.

Continuous positive airway pressure (CPAP) is indicated for moderate to severe cases of OSA. A continuous pressure of warm, moistened air is delivered through a nasal or oro-nasal mask, preventing airway collapse.^14^ Side effects include nose bleeds, paranasal sinusitis, nasal bridge sores and problems sleeping due to the bulk of the apparatus and operating noise.^15^ In fact, only half of patients persevere with CPAP therapy.^16^

Surgery to increase the volume of the upper airway has poor post-operative improvements in AHI score and carries considerable post-operative morbidity.^17^ The National Institute for Health and Care Excellence guidance on the treatment of OSA does not routinely recommend surgery due to a lack of evidence demonstrating its effectiveness. Tonsillectomy may be considered in individuals with large obstructive tonsils and a body mass index of less than 35 Kg/m².^18^

Oral appliances are the first-choice management option for mild and moderate OSA cases, or when patients with more severe apnoea don't tolerate CPAP. They are simple to make, non-invasive and cost-effective. There are a variety of designs, including soft palate liners, tongue retaining devices and mandibular advancement appliances (MAAs) (also known as a mandibular advancement device or mandibular repositioning device).^19^

## Mechanism of action of MAAs

MAAs hold the mandible in a protruded position which increases the volume of the upper airway and reduces the collapsibility of the soft palate. The tongue is also brought forward as a consequence of its mandibular muscle attachments, which opens the posterior airway and prevents it from falling onto the back of the throat creating an obstruction.^20^ These actions reduce ventilation obstruction during sleep.

Vertical opening does not affect the efficacy of MAAs and should be minimal to aid comfort.^21^ Excessive vertical opening is counter-intuitive, as it encourages rotation of the mandible in an inferior and posterior direction, which is mirrored by the tongue and soft palate due to their muscle attachments, both of which narrow and reduce the volume of the patient's airway.^22^ MAAs significantly increase nocturnal oxygen saturation and decrease AHI scores, although not to as greater an extent as CPAP therapy.^23^

## The patient pathway at King's College Dental Hospital

Patients are referred to a restorative new patient clinic by respiratory medicine with a confirmed sleep apnoea diagnosis and symptoms. A thorough extraoral and intraoral soft tissue examination and oral cancer screen should be completed, as this may be some patients only contact with a dental healthcare professional.

If dental pathology is noted during routine examination, patients should be informed and encouraged to see a dentist in primary care. However, it is the authors' opinion that patients should not be denied MAA treatment if they choose to decline this advice, as untreated OSA is associated with significant morbidity and potential mortality. If a patient does have a general dental practitioner, they should be written to with the patient's required treatment and asked to write back to the MAA provider once the suggested treatment has been completed.

## Clinical stages for constructing a monobloc mandibular advancement appliance

This article considers the single monobloc MAA design, which is, in effect, two occlusal soft splints stuck together. Alginate impressions are taken of the maxillary and mandibular arches. It is important to capture the occlusal surfaces of all erupted teeth in the impression to avoid under-extension of the splint on the occlusal surfaces and risk over-eruption of unopposed teeth.

**Fig. 1:**
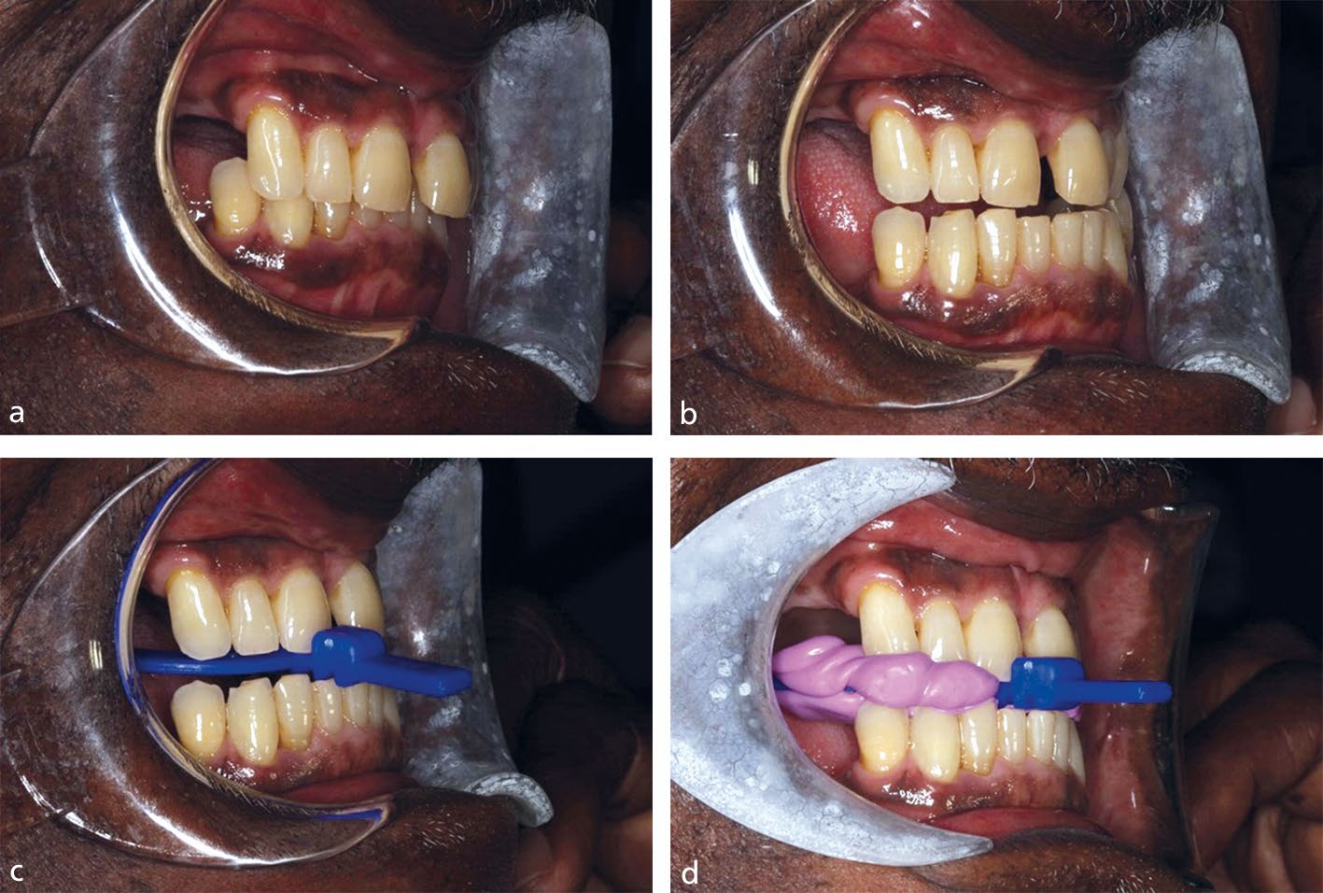
a) Intercuspal position. b) Mandibular protrusion without straining. c) Practising using the orthodontic bite fork, recording the patient's protruded jaw position. d) The final jaw registration using silicone bite registration paste to record adjacent tooth contacts to help the lab articulate the models

The patient's reproducible intercuspal position is identified, then the patient is instructed to practise posturing their lower jaw as far anteriorly as possible without placing strain on their muscle of mastication or temporomandibular joint. The amount of mandibular protrusion to make an MAA is around 75% of the patient's maximum protrusion. For most patients, this is approximately 6 mm and slightly in front of an edge-to-edge position^24^ (Fig. 1b).

An orthodontic bite registration fork is tried into the mouth and the patient is asked to bite normally onto it. The groove which the lower incisor teeth sit in is recorded. The patient is then asked to bite onto the bite fork while posturing anteriorly and the groove that the lower incisor sits in is recorded. This process is then repeated with silicone bite registration paste (Fig. 1).

If the patient easily moves from intercuspal position into a suitable protrusive position, a routine intra-occlusal record with bite registration silicone alone is suitable.

## Bite registration

**Fig. 2:**
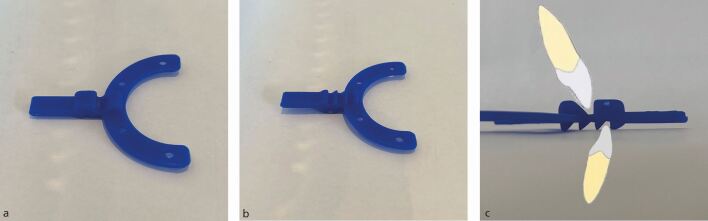
a) Superior fitting surface showing the groove for the maxillary incisor. b) The inferior surface and lower incisor grooves. c) Side profile of the bite fork showing the three potential positions the lower incisors can be seated into for different amounts of protrusion

Many methods have been described in the literature for recording mandibular protrusion. There are several advantages with using an orthodontic bite fork (Fig. 2) in conjunction with silicone bite registration paste. Vertical mouth opening is consistently kept to a minimum. You can objectively measure mandibular protrusion, depending on how many grooves forward the incisors have moved: one groove forward is 5 mm of protrusion, two grooves forward is 10 mm (Fig. 2c). By documenting in the notes which groove has been used, patients who are sent back to restorative clinics for replacement MAAs or for a greater degree of protrusion, have a historic reference to work from. The bite fork also holds the mandible in the protruded position while the silicone registration sets. This is something you do not have control over if you only use silicone registration material on its own and there is a risk of the patient unintentionally moving their jaw back into a less protruded position.

Bite forks work well for incisal Class I or II cases, but not as well for Class III cases, as the patient's lower incisor may already sit in the most anterior position on the bite fork. If this is the case, a different method of bite registration should be used, such as levering the mandible forward with a wooden spatula.

## Laboratory stages

Once received, the alginate impressions are poured in Type 3 gypsum. The casts are examined for blebs, drags or under-extensions. Light-cured resin can be used at this stage to block out undercuts or interproximal areas of periodontally involved teeth. This ensures that, when the splint is being removed from the patient's mouth, it does not engage any undercuts and place excessive force on teeth, which may exacerbate underlying periodontal issues.

**Fig. 3:**
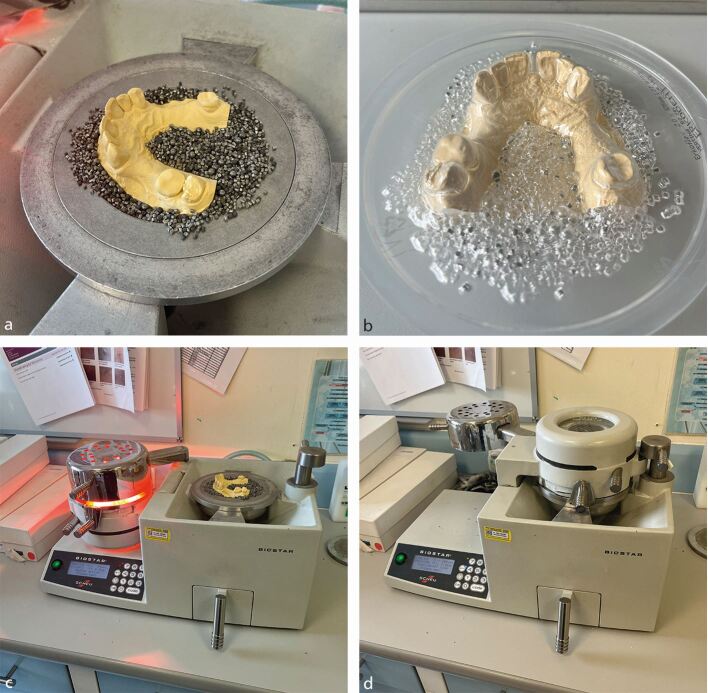
a) The casts are embedded in stainless steel granules to avoid stretching of the material during push down which would stretch the material and result in occlusal thinning. b) Untrimmed, maxillary splint. c) Thermosoftening material being pre-heated to its working temperature. d) The softened disk cooling off after being pushed down onto the maxillary cast

A thermosoftening plastic, such as polyvinyl acetate polythene, is used to make the push down splint. The product in Figure 3b is made by Erkodent and is 2 mm in thickness. The disk has a separating film applied to the fitting surface of the material to stop the material sticking to the gypsum model. This is removed at the end of splint construction. The disks of polyvinyl acetate polythene are heated to 220 °C and the material is pushed over the gypsum model (Fig. 3c, Fig. 3d).

After a 90-second cooling off period, the cast is removed and the excess material trimmed from the cast with a hot Lecron instrument. The flanges of the splint should extend approximately 2 mm onto the gingival tissues to aid retention, while avoiding mobile structure, such as frenum.^24^ The sharp edges are smoothed (Liskosil polishing bur is good for this).

**Fig. 4:**
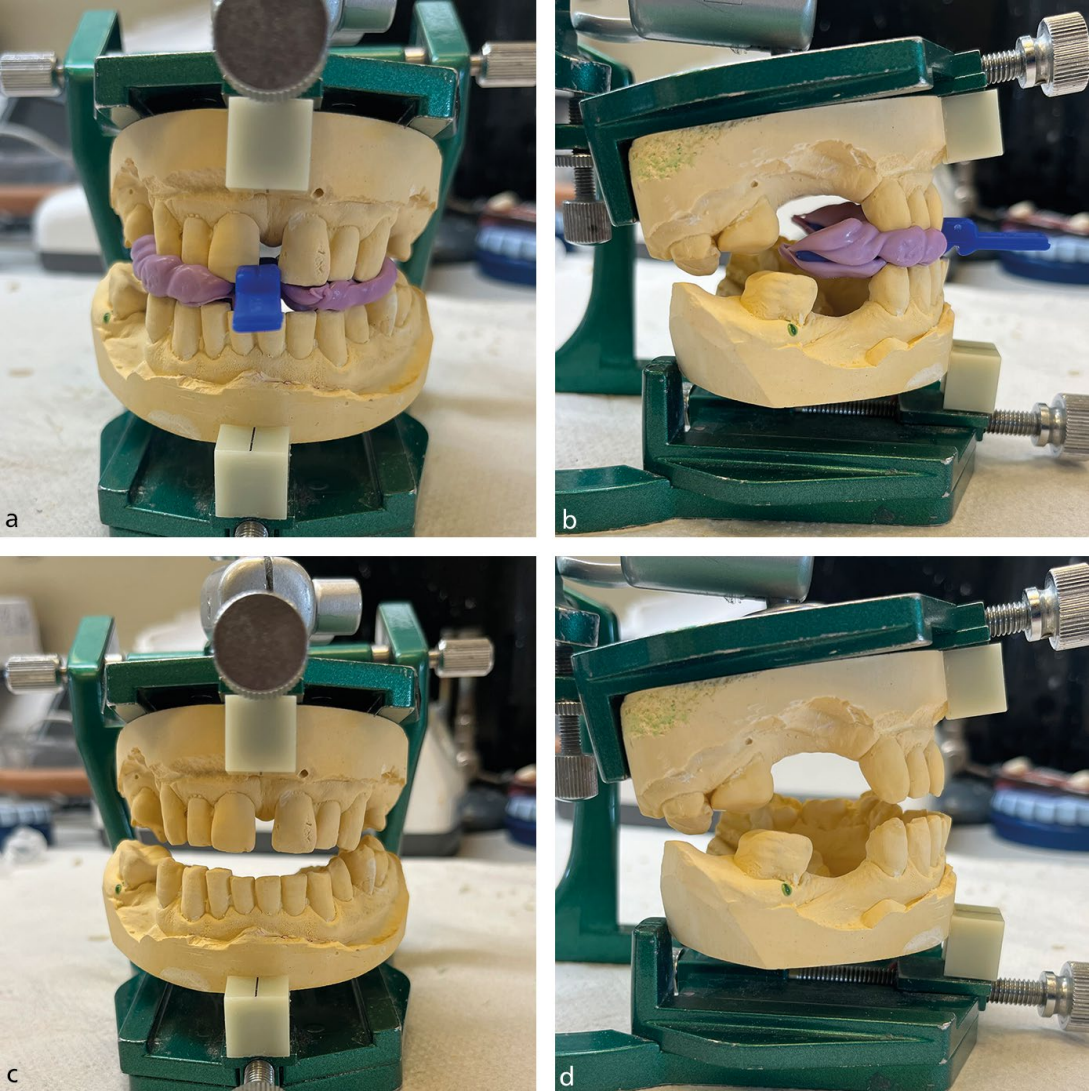
a) Maxillary and mandibular models held in position with the bite registration. b) Side on view of the mounted casts. c) Front on view of mounted casts. d) Side view of mounted casts

The models are then mounted on a plasterless articulator. The orthodontic bite fork is used to position the casts (Fig. 4).

**Fig. 5:**
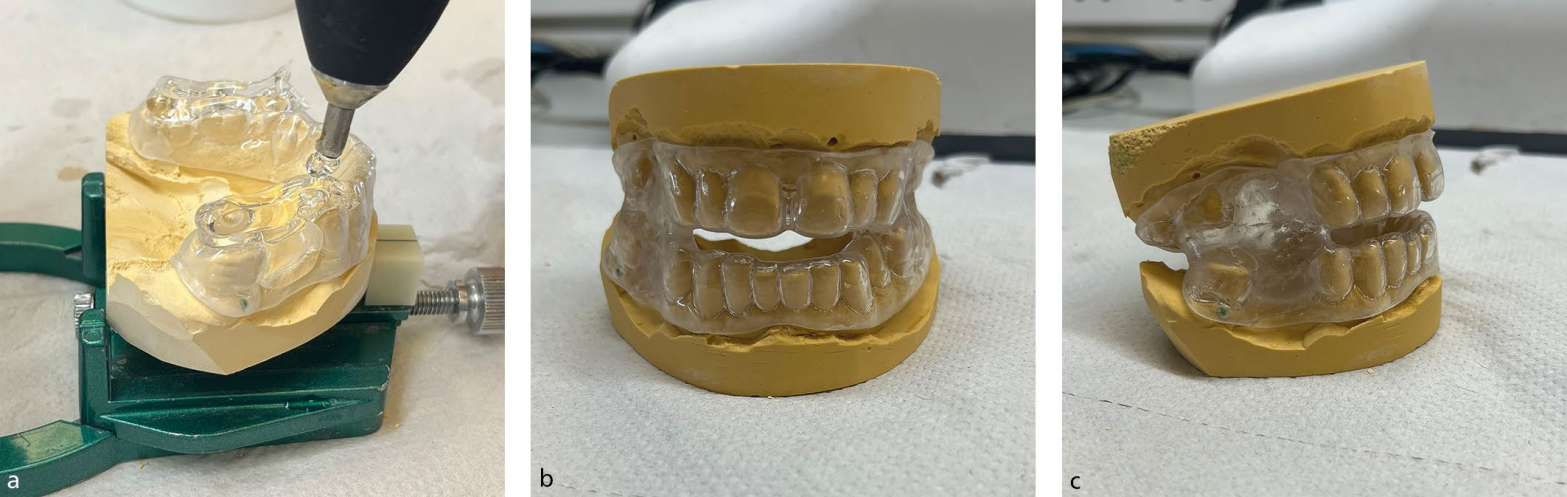
a) Hot glue being applied in posterior areas before the casts are brought together. b) Side view of added glue. c) Front view illustrating the space left for sufficient airflow

A glue gun (Steinel Professional) is used to fuse the mandibular and maxillary splints together bi-laterally in posterior areas, ensuring to leave adequate space anteriorly for airflow (Fig. 5a). The glue used (Erkodent) is made of the same material as the splint and therefore adheres to the maxillary and mandibular portions. Excess material is trimmed with a tungsten carbide bur before the added material is gently torched with a pin flame to remove any rough surfaces (Fig. 5b, Fig. 5c).

## Fit appointment and aftercare

At the fit appointment, insertion and removal of the device is demonstrated. The maxillary portion of the splint is inserted first and then the patient is instructed to bite their teeth into the lower portion. Patients are advised to wear the device at night and to only insert the device after cleaning their teeth to avoid food being in contact with the teeth overnight. The device should be disinfected regularly (Steradent) and hot water should be avoided, as this can deform the appliance as it is thermosoftening.

The patient is reviewed after eight weeks with respiratory medicine, where a new ESS and API score is recorded.

## Commonly reported problems

Side effects of MAA therapy include increased salivation, unpleasant tastes, nausea and exacerbation of symptoms of underlying temporomandibular joint disorders. These side effects are transient and part of the adaptation process. MAA compliance is higher than with CPAP.^25^ Only one patient out of several hundred treated at King's College Dental Hospital has reported separation of the maxillary and mandibular splints.

The splint prevents saliva from reaching the teeth and can trap food around the teeth, increasing the risk of dental decay and periodontal disease. The importance of good oral hygiene must be stressed. A study concluded prolonged use of adjustable repositioning devices increased the risk of adverse occlusal changes, such as mesial migration of the lower dentition and distal migration of the upper dentition.^26^ Further investigation is required into the long-term effect of MAAs on the dentition.

## A multidisciplinary approach

The potential sequelae of untreated sleep apnoea include increased risk of cardiovascular disease, stroke and nocturnal mortality.^27^ Dental Protection Limited published guidance on OSA for dental practitioners, stating that patients with suspected OSA should be referred for medical assessment and officially diagnosed by a suitably trained specialist before an appliance is made, as OSA diagnosis lies outside a dental practitioner's scope of practice.

Dental Protection also recommends that dentists undergo a documented training course, which includes training on screening for OSA before providing appliances.^28^

Making a patient an anti-snoring device may mask the symptoms of sleep apnoea, leading to a delay in its diagnosis. Being aware of the signs of sleep apnoea allows dentists to identify and appropriately refer at-risk patients for appropriate medical assessment.

## Conclusion

The awareness of OSA is increasing within the healthcare profession and the potential role of dental practitioners in its treatment is an emerging field. The dental profession is in a unique position to work with medical professionals in providing an integrated treatment plan to provide patients with a simple, cost-effective device, which can dramatically improve quality of life.
